# Integrating Heterogeneous Biomedical Data for Cancer Research: the CARPEM infrastructure

**DOI:** 10.4338/ACI-2015-09-RA-0125

**Published:** 2016-05-04

**Authors:** Bastien Rance, Vincent Canuel, Hector Countouris, Pierre Laurent-Puig, Anita Burgun

**Affiliations:** 1 University Hospital Georges Pompidou, Paris, France; 2 INSERM UMR_S 1138, CRC, Paris, France; 3 Université Paris Sorbonne Cité, Inserm UMR-S 1147, Paris, France

**Keywords:** Data integration, medical information systems, translational medicine, translational research platform

## Abstract

Cancer research involves numerous disciplines. The multiplicity of data sources and their heterogeneous nature render the integration and the exploration of the data more and more complex. Translational research platforms are a promising way to assist scientists in these tasks. In this article, we identify a set of scientific and technical principles needed to build a translational research platform compatible with ethical requirements, data protection and data-integration problems. We describe the solution adopted by the CARPEM cancer research program to design and deploy a platform able to integrate retrospective, prospective, and day-to-day care data. We designed a three-layer architecture composed of a data collection layer, a data integration layer and a data access layer. We leverage a set of open-source resources including i2b2 and tranSMART.

## 1. Introduction

Cancer research ranges from epidemiology, basic research and molecular bioscience to translational research and analysis of routine care data used to evaluate and compare applications of the various therapies. Similarly, cancer care also involves multiple fields including ‘omics’ sciences, such as genomics. High-throughput technologies are indeed now part of the everyday care in most hospitals [1, 2]. For example at the European Hospital Georges Pompidou (HEGP), the identification of genetic variants through targeted gene sequencing is common practice for cancer patients, to guide physicians in the choice of the best treatment.

Concurrently, the increasing adoption of EHRs worldwide provides a steady flow of structured clinical data for care and research. Major medical research centers such as Vanderbilt [3] or Harvard [4] have demonstrated the research opportunities opened by secondary use of data. The global movement toward an increased secondary use of care data opens new perspectives for researchers and clinicians, and raises new technical and ethical questions [5].

However, the adoption of integrated translational research platform in healthcare institutions has been slow, partly because the problem is still complex, both from the technical perspective (e.g. there is often no shared identifier and no common data model), and from the regulatory standpoint [6]. Therefore, the construction of an integrated platform is still a major challenge. In [6], the authors described the results of a 2010 survey by the CTSA (Clinical and Translational Science Award) consortium’s Informatics Integrated Data Repository Group. The survey identified major obstacles to Integrated Data Repositories, including data access, data quality and standardization. The article also noted that there was a movement away from ‘home-grown’ systems toward more commonly used systems (such as i2b2 [7, p. 2]). Among the obstacles, data access was listed as an obstacle for project approval and a continued difficulty. Paradoxically, the public acceptance of such data repository is overall fairly positive. A survey from 2013 by Grande et al. [8] investigated the public preferences toward secondary uses of electronic health information. The main concerns identified were the use of the data, before the data sensitivity and the user. The survey showed a general willingness to share health information for research use, especially in the context of research hospitals.

In this article, we describe the solution adopted by the CARPEM cancer research program to design, implement and deploy a translational research platform able to face the technical and scientific challenges, and the issues underlined by the CTSA survey.

### The CARPEM program

The French government introduced in 2009 a 4-year plan aiming at strengthening the resources dedicated to cancer research. This plan recommended the creation of a competitive policy label to ensure the organization and management of interdisciplinary research and knowledge dissemination programs. In 2012, the French National Cancer Institute (INCa) granted eight SIRICs (*Site de Recherche Intégré sur le Cancer* in French, or Integrated Cancer Research Site) labels in France. SIRICs’ ambitions are to provide new operational resources to oncology research, to optimize and accelerate the production of knowledge and to favor knowledge dissemination and application in patient care.

The CARPEM (CAncer Research and PErsonalized Medicine) program is one of these eight SIRICs, focused on selected tumor types: digestive, endocrine, head and neck, hematological, lung, ovarian, and renal tumors.

The CARPEM program develops the following cross-disciplinary research objectives leading to the development of personalized medicine: (a) identification of genetic- and immune-based prognostic and predictive biomarkers with the help of translational project and epidemiological cohorts, (b) development of experimental therapeutics, and (c) integration of ethics and assessment of practices in the CARPEM research program.

The CARPEM Translational Research Platform is a transversal project working with all the themes and tumor localization of the CARPEM research program. The platform aims at collecting and integrating all data related to cancer patients from the participating clinical and research institutions. The goal is to provide researchers with a complete picture of the expression of the disease, in terms of comorbidity, biology test results, genomic data, and so forth.

Data collected are heterogeneous in their nature and their semantics: structured, partially structured and unstructured clinical data of various data-types and various levels of granularity (from molecular biology analyses to phenotypes). CARPEM aims at collecting a large array of data, including: (i) phenotypic structured data, including routine care records and Clinical Trial observations, outcomes and adverse events; (ii) high content biomarker data, such as gene expression and proteomics data; (iii) unstructured text-data.

Data are produced and stored in various places: three participating hospitals (the HEGP, the Cochin Hospital, and the Necker Hospital), research laboratories from many different specialties (several geographical localizations). Within each site, the information systems and software are heterogeneous and there is no unique patient identifier across the CARPEM data sites.

Within the institutions, the data media are heterogeneous. Retrospective data often come as text or Microsoft Excel spreadsheet, more rarely as organized relational databases. Clinical data are collected from institutional Clinical Data Warehouses (CDWs), from dedicated software (chemotherapy management software…), or from text-based clinical reports. Finally prospective data are fed to the system through Clinical Data Management Systems. The data collection process highly depends on the type of data: data collected in the context of a clinical trial have been strictly controlled and curated at different levels; whereas routine care data may exhibit missing information.

### Related work

Platforms enabling an integrated view of clinical and ‘omics’ data have received lot of attention in the recent years. Platforms of every size, capacities and goals have been developed [9]. For example, projects such caBIG [10] were designed as early as 2004 for multiple purposes, including to enable data sharing at a U.S. national scale. However, this project is not suitable for the exploration of local data-sets, or as a local data integration solution. Alternatively, standalone platforms appeared (e.g. iCOD [11], BRISK [12] or tranSMART [13]). These platforms enable an efficient exploration of the data. They are usually composed of a database, and an application server. These solutions are reasonably easy to install, and might also be shared in virtual appliances. Finally, research centers have deployed translational research platforms to allow data integration, cohorts selection, hypothesis generation and data-exploration [3, 14–18]. These platforms are deployed at an institutional or regional scale and often integrate de-identification, patient identity management in addition to the integration and exploration functionalities.

CDWs have also been adapted to handle the new types of data generated by Next Generation Sequencing and other high throughput technologies. For example, the i2b2 (informatics for integrating biology and the bedside) [7, p. 2]was extended to store and manage data from biobanks as well as free-text [19].

Complex integration platforms share similar functionalities; namely, data collection, format integration, de-identification, semantic integration, making data available from researchers to browse and explore. ETL processes (Extract, Transform, Load) are often a large part of the integration workflow to capture, format and transport the data. Two open-source solutions are often used (Pentaho Data Integration or Talend Open Studio). In many project, the semantic integration is performed through ontology mapping [20, 21], a domain that received and still receives a great deal of interest [22] with a dedicated yearly workshop [23].

## 2. Material and Methods

In this section, we will describe the CARPEM environment and the method used to develop the platform.

### Datasets

The CARPEM program integrates both general university hospitals with a strong activity in oncology and oncology-related care, and research centers. These institutions generate a large range of data types, going from clinical data collected during day-to-day care to data type at the forefront of modern techniques. The platform integrates data from patients previously or currently treated for a cancer in one of the participating clinical institutions (namely, the Cochin Hospital, the HEGP, and the Necker Hospital).

There is no uniform consent collection within the CARPEM program. For samples collection, the clinician relies on informed consent. The secondary use of care data leverages an opt-out solution. The overall data collected by the platform can be categorized as follow:

- Clinical data from the clinical care systems
- Retrospective data from clinical research groups (e.g. from CRF)
- Research data from associated labs
- ‘Omics’ data from associated molecular labs
- Environmental data (e.g. profession)
- Additional sources (e.g. data from biobanking)

The data environment is expected to evolve over time. New biological methods are developed (in immunology for example), and will need to be integrated in the platform.

### Open-source and community-based software and components

The development of a translational research platform is a costly process. Creating specialized tools for data collection or data exploration is complex both in the initial development and for their maintenance over time. On the other hand, the community has developed over the years many high quality resources for all these tasks. We first identified our needs: an eCRF for prospective data collection, ETL (extract, transform, load) processes, data storage and data exploration; and we reviewed the community tools available and selected the ones best fitted to our needs. The reviewing process was performed through a formal literature review [9], and previous experiences. We finally leveraged six tools and format described below.

*REDCap* [24] is an electronic Case Report Form and a Clinical Data Management System developed by the Vanderbilt University and partially funded by the U.S. NIH. It is used by internationally by a broad community of users in institution of various sizes.

*i2b2* “Informatics for Integrating Biology and the Bedside” [7] is an NIH-funded National Center for Biomedical Computing (NCBC) based at Partners HealthCare System in Boston. It provides an infrastructure for CDWs, which has been adopted by numerous academic hospitals around the world [25–27]. The i2b2 warehouse uses an Entity-Attribute-Value (EAV) data model for its adaptability and dynamic nature; concepts are stored separately in a hierarchical data model.

Prior to the development of the platform, we had conducted a review of the biomedical literature to identify translational research software allowing the combined exploration of clinical and omics data [9]. In this review, we compared seven platforms on seven axes (including types of data handled, existence of a community of users). We decided to adopt tranSMART. *tranSMART* [13] is a translational research platform allowing the integration of clinical data and omics data. It provides exploration and analysis tools. The tranSMART platform is developed by an active open-source community. We use the 1.2 PostgreSQL version of tranSMART.

*Talend Open Studio* [28] is an open-source data integration software allowing a graphical design of ETL pipelines.

*FreeMind* [29]is a free mind-mapping tool. It allows user to “edit a hierarchical set of ideas around a central concept” [30]. We leverage the software strength to easily edit trees of concepts.

*CDISC ODM* [31]: We also leverage a community based format from the CDISC consortium. The CDISC ODM standard is designed to represent clinical studies, and to enable the storage of clinical data.

### Harmonization process for prospective data

One of the goals of the CARPEM program is to stimulate data sharing and collaboration among researchers. CARPEM researchers are organized into working-groups by localization of the tumor: lung cancer, ovarian cancer, colon cancer, and so forth. The task of the working-groups is to harmonize the data collection process among the participants of the group.

*Seek and describe available data.* Each group identifies existing retrospective datasets, and describes them (in terms of variables, number of patients, time period covered by the dataset, produced publications, etc.).

*Establish minimum shared datasets.* In parallel, the working group constitutes a prospective cohort by determining a set of shared variables. We first establish semantic mappings between the items collected by the different groups. We identify common items shared through the groups, and items used only by a limited number of research groups. We then select a set of item by consensus method. This step is complex and time consuming. Finally, We filter out the establish list to keep only a *minimum* set of items.

*Standardize data collection tools.* This step demand a significant amount of work for clinicians as it requires an evaluation of the clinical care collection tools. To summarize, we build a data dictionary defining preferred terms for each item, units, how the information should be structured and formatted. When possible, we identify mapping to concept in standard terminology.

This task is highly time consuming, but allows sharing a common understanding of collected data, and has strong clinical implications.

### Security and technical implementation of the platform

The platform runs on 7 Linux servers: three production servers (one dedicated to databases, the other two to applications), two development servers (one dedicated to databases, the second to applications), one data-integration and record-linkage server and one data-capture server. Servers have AMD Opteron 6320 processors (8 cores, 2.8GHz) and RAM ranging from 16 to 64 GB. The total disk space dedicated to the infrastructure is 14TB. In addition to the use of RAIDs for system and data, backups of data and systems are made on a regular basis on a NAS. In-house development is Java-based, user interfaces are web-browser accessible.

The platform is hosted within the network of the HEGP, and beneficiates from institutional firewalls from the HEGP and from our parent institution the AP-HP. Within the institution, access to servers handling identifiable data is restricted to the platform personnel only.

### Requirements

The underlying goal of the CARPEM platform is to enable easy data-sharing and data-exploration for clinicians and researchers, through state-of-the-art data representation and organization. The platform should also be able to integrate data from several sources, and enable a global view on the patients and their disease. To achieve these goals, we defined a series of scientific and technical requirements to guide the design and development of the platform.

### Scientific requirements

#### A hypotheses free platform

The design of the platform cannot be tailored to a specific condition.

#### A centralized patient management

Our project integrates data from several institutions, which do not share a common patient identifier. The platform has to be able to manage patient identities across the program and should provide de-identification services to be able to generate anonymous data files for research.

#### A centralized ontology/terminology management.

The CARPEM data are coded to several terminologies, such as ICD (International Classification of Diseases), or ATC (Anatomical Therapeutic Chemical classification). Semantic interoperability must be achieved, and terminologies have to be managed to allow interoperability (e.g. through across-study analysis), or efficient query management (for example, through terminology-based query expansion).

#### Provide data with metadata and annotation

Data sets are reliable within a collaborative project when their provenance, i.e. the process used to create them, is captured and stored. Data need to be stored along with metadata detailing how they were generated, and the semantic they hold. Data stored in the platform must be annotated with standard vocabularies (such as SNOMED CT, the Gene Ontology, or the Human Phenotype Ontology) to allow analysis of associations between phenotypic and biomarker data.

#### Data sharing and data-property

Effective data property management is important to gain the trust of users, and of the public. Many researchers would welcome new and modern data exploration tools, but not at the price of publicly sharing data that they obtained at the cost of hard work and manpower. To convince users, the platform must implacably handle data-property.

#### Ethics and policy

In addition to issues related to sharing data safely among the different stakeholders, several ethical issues related to secondary use of patient data must be addressed: the content of informed consent, patient awareness and acceptance regarding how their data are used and shared (especially ‘omics’ data), and the respect of patients’ rights regarding their access to and control of their data. One difficulty of ethics management with large data warehouse is the lack of extensive regulation and policies in France.

#### Manage several complementary levels of knowledge granularity

A translational research platform handles a variety of users with various expectations on what kind of information the platform should be able to provide. For example, regarding *Single Nucleotide Polymorphisms*, a clinician might want clear presence/absence information, which can be used to make decision about the treatment, while a bioinformatician or a biologist might ask for a more detailed picture.

### Technical requirements

#### Data security

The first concern developing an integration platform must be the security of the patients’ data. Security must be state-of-the-art (encryption of communications, access policy…), and must include several layers of protection. Clinical and research identifiers must be protected against confidentiality breaches, possible attacks, or unfair use. In addition, data must be protected against accidental loss.

#### Data Quality control

One main difference between clinical research data and care data is the amount of time spent appraising the quality of the information. In clinical research, the data is assessed by clinical research associate, and curated by data-managers. Care data do not undergo such process. We aim at providing the researchers with controls and management-plan for clinical care data through the detection of missing data or outliers, and consistency checks.

#### Modularity

Technology in the field of molecular biology and medical informatics is changing fast. New algorithms and technics appear on regular basis. Our platform needs to be able to handle new data format and new results. Modularity is key to keep the platform up-to-date.

#### Leverage community-developed components and solutions

The community spends a tremendous amount of work developing high quality, high performance tools. In the CARPEM platform, we wish to limit in-house development as much as possible. Required enhancements of existing tools and software will be shared with the community.

#### Scalability

The platform has to handle small datasets as well as large cohorts and genomics data. The loading, management and storage processes have to be able to handle several million data-points.

#### User management

Due to the sensitivity of the data, and the complex question of ownership of the data, the platform has to be able to identify at all-time who has accessed, or had access to the data. Logs of data use are needed. Proper user management dealing with privileges needs to be in place.

#### A unique standard exchange format for clinical data

All inputs have to undergo the same set of transformation (namely: record-linkage process, de-identification, and data-quality control), despite the wide heterogeneity of input formats. To avoid locally tailored solution, all data should transit to the platform using standard representation based on open format (such as CDISC or HL7).

## 2. Results

### Architecture of the platform

**Fig. 1:**
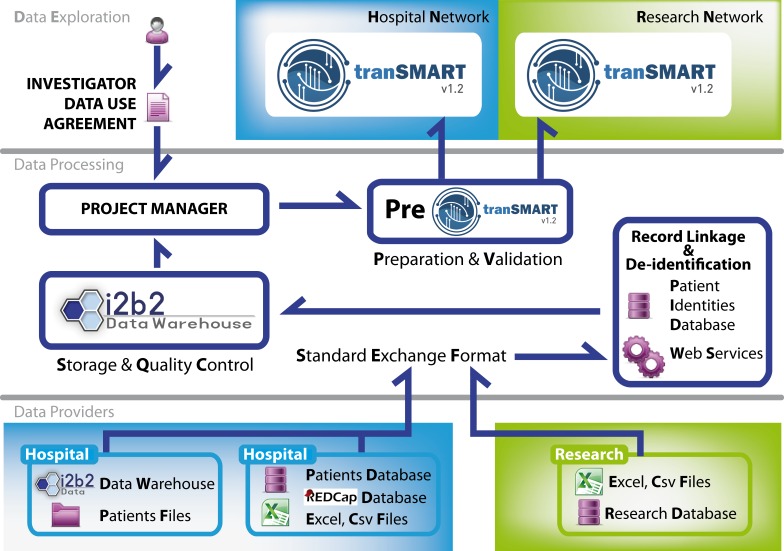
CARPEM Translation Research Platform Architecture. The bottom layer represents the data providers (namely, hospitals and research labs). Locks represent controlled access: at institutional levels (represented in blue), or during the data integration (on arrows).

This next section gives an overview of the platform. ► Figure 1 presents the three-layered organization of the platform. To summarize, the architecture of the platform is the following: data are collected or captured from heterogeneous sources in the first layer; they are standardized into a common exchange format. In the Data Integration layer, all record-linkage and de-identification processes are handled, and data are stored. Finally, data are made available to researchers in the data access layer.

### Data capture and data source layer

#### Data extraction and curation

Data are collected either through a secured file exchange system, or directly extracted from the information system within the CARPEM program institutions. Hospitals and research institutes generate two types of data: (1) structured data (e.g. billing code, relational databases, Case Report Form…) and (2) unstructured data (mostly free-text reports such as discharge summary or imaging reports). As of today, the platform does not manage free-text reports. However, information contained within unstructured data is very valuable. We therefore developed specific parsers to extract structured knowledge from free text. Parsers are based on the structure of the documents and are dedicated to specific tasks. We will continue to develop such tools on a study-based basis.

One difference often highlighted by some authors between clinical research data and secondary use of care data is quality [6]. During a clinical trial, data are controlled at virtually every step of their life cycle. Whereas, raw clinical data do not beneficiate from any data-management.. To ensure the best quality possible, the translational research platform team performs data quality control to detect issues in datasets. More specifically, data are checked for different types of errors including missing information (including identification of the patient and description of the disease: localization, grade…) and consistency (e.g. chronology of dates). For retrospective datasets, the team optimizes the data representation to insure that data are leveraged to maximize their intrinsic value. *Data representation.* The data-exploration interfaces of tools such as i2b2 or tranSMART rely on a hierarchical organization of the variables to express subsumption relationships (parent-child relations), e.g. to navigate, or to expand queries. When possible, we leverage standard medical taxonomies. However, for specific studies, if the clinician needs to maintain the organization of the data, we leverage FreeMind [29] to build a model of a local terminology. An item can be assigned to one or more concept. A study can therefore be associated with a local terminology as well as with a standard terminology.

#### Standardization of the exchange format

Despite the heterogeneity of input formats (e.g. retrospective MS Excel datasets, data from the institutional CDW, relational databases), the actions performed on the data are always identical: record-linkage, de-identification, data quality control, standard storage. Instead of developing ad-hoc ETL processes for each new entry format, we chose to transform every input file to a unique exchange format based on the CDISC ODM standard [31].

The exchange ODM file is composed of 3 parts: the administrative data part holds all information regarding the provider, the date of generation of the dataset, etc. The metadata describes the structure of the variables (data definition, range, units…). Finally, the actual data are stored in the clinical data part of the document. We developed a limited vendor-extension to the original CDISC ODM format to ensure the best interoperability between all parts of our system. Our vendor-extension of the ODM model adds a notion of “Personal Identifiable Information” status (Identifying or not). The ODM model is limited in depth (with a maximum of four hierarchical levels of depth), we also leverage the reference to hierarchies (standard or local) to enable the organization of concepts without depth limitation. Because of its single central role, the creation of the ODM exchange file is critical to the rest of the process. Both the data-managers and the data-provider (researcher or clinicians) are involved in this step. The ODM exchange file metadata provides us with a data dictionary describing at a high level the content of the datasets.

### Data integration, storage and management layer

An ETL process takes the exchange ODM file in charge through the rest of the framework.

#### Data Loading

The ODM exchange files are loaded through a standardized ETL process, and will go through the following steps: record-linkage, de-identification and data storage. The ETL process uses the open source software *Talend Open Studio for Data Integration.*

#### Record-linkage

CARPEM patients (and their data) come from a variety of locations and institutions: one of the CARPEM program hospitals, molecular biology laboratories... No unique patient identifier is shared across these institutions. The platform provides a unique CARPEM identifier and attempt to link records from the different sources. We rely on first name, last name, sex and date of birth as our main identification key. Alternatively, institutional identifiers can also be used to identify patients within a given context. We leverage the Rochester Epidemiology Project[32] mapping rules, and link records through a series of decreasingly selective rules. The record-linkage is fully automated. However, all record-linkage choices are recorded, and can be later corrected if needed. Straightforwardly, the strictest rule defines that two individuals sharing exactly the same first and last name, as well as same gender and date of birth are considered to be the same person.

If insufficient information is provided for a patient, the system can create a new “non-matchable” entry. The data are de-identified for the current patient, but no future patient data will be linked to the entry. This process limits the risk of erroneous match.

Identities of the patients, their unique CARPEM identifier, as well as identifiers across the participating institutions, are recorded in a relational database. However, the interaction between the platform and the patient database is performed through a web service, limiting the risk of mistakes during the data processing (only a strictly limited number of functionalities are allowed through the web service).

#### De-identification

The French law does not provide a list of identifiable variables that should be removed from patient data to ensure anonymization. We used the HIPAA Safe Harbor [33] recommendation as a guideline. For each patient, we remove all directly identifying data and we choose a random number between -365 and 365 to shift all dates. The date-shifting value is stored in the patient database. Identifiable data and clinical data are kept in separate databases installed on separate servers.

#### Data storage and management

We leverage the i2b2 platform [7] to store our data. The i2b2 data model is composed of 6 main tables organized in a star schema. For each new record, we collect and store information on the provider (who generated the data) and the provenance (the exact context of the data generation), as well as detailed information about the measured variables.

Data that cannot be efficiently stored in the i2b2 format (e.g. images or gene expression data) are stored as references to files on our local file system or on the hospital information system.

Not all data are accessible to all users. The platform manages transversal projects as well as project managed by a small group of researchers. In the CARPEM translational research platform, data and data-access are handled by “project”. We define a project as a set of concepts, for a set of patients accessible to a set of users.

### Data access layer

For the final users of the platform, researchers and clinicians, the data-integration layer is a blackbox. The only visible part is the data access layer. We provide tools to the users for the exploration and data-driven hypothesis generation.

We leverage tranSMART to allow clinicians and researchers to explore their data, without complex interaction with bioinformaticians and biostatisticians. TranSMART allows data exploration through a web application as well as data interrogation through an API. It can be used by users with programming skills as well as without any expertise in computer science. Additional software and tools will be deployed in the future to respond to the users need.

### Ethics, legal and data-property issues

#### Ethics

Ethics is a core concern of the construction of the platform. The CARPEM program adopted a dual solution. Data collected during the care process are integrated under an opt-out consent policy. Patients who wish not to be included in research projects can express their request at any time during or after their visit at one of the hospitals. For research data (retrospective, prospective, ‘omics’ and so forth), an opt-in solution has been adopted. Users of the platform (i.e. clinicians and researchers) have to accept a user-agreement, in which they are reminded of basic ethical and common-sense principles: respect of private information, use the data in a secured environment, interdiction of patient re-identification, interdiction to share their personal identifiers.

#### Data property

Datasets are classified in two categories: public and private datasets. Public datasets are shared without restriction. Private datasets are accessible only to the project’s PI by default. The PI or a mandated member of the data-provider’s team may give access to all or part of a datasets, to one CARPEM researcher or a group of CARPEM researchers. To be eligible for data-sharing, researchers need to have complied with official obligation, and to have accepted CARPEM data user agreement.

For retrospective datasets, the identification of data ownership is clear. For clinical data from hospitals, ownership is discussed within the clinical working groups, prior to the collection. By default, the clinical teams implicated in the collection of the data are considered owners.

### Adoption

The CARPEM translational platform is young. Out the eight working groups by cancer localization, only four have started the identification of shared minimum datasets, and the integration of data in the platform. Moreover, the platform has been mostly dedicated to the integration of retrospective datasets during its first year. Five clinical and research groups have contributed 13 datasets. Six datasets have been loaded into tranSMART, three in i2b2, representing a total of 5,700+ patients, and a little over 300,000 database records (including 24,500 diagnosis, 19,900 procedures and 57,300 biobank items). The REDCap eCRF software currently handles 10 ongoing studies.

The integration of data streamed from hospital IT is at an early stage of development and includes billing codes (ICD10 data and normalized procedures).

The data integrated range from clinical data, genomics (including mutations obtained by NGS, copy number), immunological data, to biobanking data.

Current projects involve physicians and researchers from several departments, including Molecular Biology, Gynecological Surgery, Pulmonary Surgery, Immunology, and Pathology.

## 4. Discussion

### Expected benefits

The objective of data sharing has already led to harmonization efforts. Clinicians and researchers who plan to pool their data and increase the statistical power of their studies, thanks to the CARPEM platform, have defined common data elements that will be collected in prospective data sets. Data harmonization efforts concern all items, including clinical phenotype descriptions, surgical reports, imaging reports, treatment and follow-up, genetic data, and adverse events. The meetings dedicated to data sharing tools will facilitate and support the continuous process of harmonizing the data collection among the participating hospitals within the CARPEM program. By providing efficient data management and user-friendly exploration tools we hope to generate even more research hypotheses. Finally, the platform enables data integration that was out of reach until now. Phenotypic enrichment with any type of data is now possible and easy to set up.

### Technical significances

#### Modularity

The CARPEM translational research platform is at an early stage of its development. We plan to include progressively more and more data sources as well as new types of sources such as connection to biobanks. Our model eases the addition of new sources of data. The only need is to develop an ETL process to transform the initial source into the ODM exchange file.

#### Compatibility

By leveraging as many community-based solutions as possible, we ensure an easy interoperability with other users and with other platforms as well as the ability to replicate studies done by other groups. We will also beneficiate from initiatives, and will be able to share local development and methods.

#### Community involvement

Among the eight SIRICs, five are developing data platforms. The i2b2/tranSMART technical choice has been adopted or is being explored by two groups in addition to the CARPEM program.

#### Generalization

The CARPEM Translational Research Platform was designed for cancer research. However, only a limited part of the components are dedicated to the topic (including free-text parser). The architecture could be transposed with little effort to other medical domains.

### Remaining challenges

#### Semantic integration

The Translational Research Platform is not yet equipped with a central management for ontologies. As the number of datasets integrated increases, it appears crucial to beneficiate from such functionality.

#### Free-text

A large part of the clinical information is still buried within free-text reports. This knowledge is especially important as it often covers information that is usually not present as structured data, such as the history of the disease and the episodes of care outside of the hospital. A large body of work can be found on the use of natural language processing (NLP) of medical text, but efficient extraction method that would take into account temporal relations is still an open problem, especially for non-English languages.

#### Federated query

The architecture presented in this article focuses on the integration of data in one single data warehouse. In specific cases the transfer of data might not be possible; e.g. because of regulations, or data ownership dispute. Federated queries might be helpful for such cases. For example, SHRINE [26] – a federated querying system for i2b2 – could be used for cohort selection, and could enable result sharing without having to share actual datasets.

### Limitations

One major problem for the analysis of both clinical and ‘omics’ data is the question of granularity of the information. A large translational platform, such as the one we aim to build, has a variety of users interested in very different level of information.

Managing the level of access for each category of users of every piece of information can become a very complex task. This is however a challenge that we will need to embrace in future version of the platform.

#### Record-Linkage

Our platform is designed to handle data integration in a semi-automatic manner. We reduced the human involvement in the processes as much as possible. More advanced methods are often possible, for example statistical record linkage, and could be performed manually on the platform. However, this has not been identified as a priority by our users.

#### Text-mining

The CARPEM translational research platform does not handle free-text. A large part of clinical information is contained only in text report [34]. Numerous Natural Language Processing techniques [35] and initiatives have been developed to make usage of this rich information. We plan to include NLP tools in our framework. However, free-text processing may raise issues, especially with regards to the de-identification of data.

### Future work

The translational research platform is at an early stage of its deployment in the CARPEM program. Although we are confident that its architecture and functionalities will help the researchers in the data exploration tasks while ensuring an efficient data management and quality control, the use of the platform for large scale cohorts remains to be assessed. Moreover, with high-throughput technologies being more and more involved in the everyday care of patients, we expect a dramatically increasing amount of data being available per patient, with an associated responsibility for the researcher to make use of such data. Big-data technical solutions (such as MapReduce, or NoSQL solution including the ADAM genomics analysis platform leveraging the Apache Spark technology) might become a mandatory step in the construction of translational research platforms in a near future. New tools exploration and analysis tools will also be provided to our users in future versions of the platform (for example, the cBioPortal [36], or the R statistical software https://cran.r-project.org/). Additions to the platform will remain guided by our set of requirements and users recommendations.

## 5. Conclusion

We designed and built a translational research platform for the CARPEM cancer research program. We evaluated the needs and requirements of translational research and outlined scientific and technical principles that guided us in the construction of the platform. Technically, the organization of the platform can be summarized as follows: we standardize inputs into a single exchange format, then de-identify the data and integrate them into an i2b2 data warehouse. We then push datasets to exploration tools such as tranSMART.
